# Serum Albumin and Circulating Metabolites and Risk of Venous Thromboembolism: A Two-Sample Mendelian Randomization Study

**DOI:** 10.3389/fnut.2021.712600

**Published:** 2021-11-11

**Authors:** Zhengye Liu, Jiarui Mi

**Affiliations:** ^1^Second School of Clinical Medicine, Zhongnan Hospital of Wuhan University, Wuhan, China; ^2^State Key Laboratory of Cardiovascular Disease, National Center for Cardiovascular Diseases, Fuwai Hospital, Chinese Academy of Medical Sciences and Peking Union Medical College, Beijing, China

**Keywords:** mendelian randomization analysis, venous thromboembolism, serum albumin, metabolic syndrome, monounsaturated fatty acid

## Abstract

**Background and Aim:** Previous observational studies indicated that the serum albumin levels and circulating metabolites are associated with a high risk of venous thromboembolism (VTE). However, whether these observations reflect causality remained unclear. Hence, we conducted a two-sample Mendelian randomization (MR) analysis to evaluate the causal associations of serum albumin and circulating metabolites with the risk of VTE.

**Methods and Results:** Summary statistics of genetic instruments proxying serum albumin, total protein, and common circulating metabolites were extracted from genome-wide association studies in the European ancestry. Summary-level results of age- and sex-adjusted estimates for associations of the instruments with VTE were derived from the FinnGen consortium. We used the inverse-variance weighted (IVW) method as the primary analysis for univariable MR. Sensitivity analyses were performed to detect horizontal pleiotropy and outliers. Genetically proxied high-serum albumin and total protein levels were suggestive protective factor of VTE, with odds ratio (OR) = 0.69 (CI 0.54–0.89, *p* = 4.7 × 10^−3^) and 0.76 (CI 0.61–0.95, *p* = 0.015), respectively. Genetically proxied low-monounsaturated fatty acids and the ratio of monounsaturated fatty acid to total fatty acid are causally associated with increased risk of VTE, with ORs = 0.89 (CI 0.80–0.99, *p* = 0.031) and 0.85 (CI 0.78–0.94, *p* = 9.92 × 10^−4^), respectively. There is no indication of causal associations between other circulating metabolites and the risk of VTE.

**Conclusions:** Genetically liability to low-serum albumin and total protein levels, low proxied monounsaturated fatty acids (MUFAs) and the ratio of MUFAs to total fatty acids are associated with the higher risk of VTE.

## Introduction

Venous thromboembolism (VTE), including deep vein thrombosis and pulmonary embolism, is the third most common vascular disease affecting 10 million cases every year and is now a major clinical burden (1, 2). The development of VTE is a complex process involving environmental and genetic factors (3). Previous efforts to prevent VTE mainly focus on the hospital-based factors such as cancer, history of surgery (4). Recent studies have shown that the circulating metabolites are also associated with the risk of VTE (5–8). Patients with hypoproteinemia are more prone to develop VTE. Recent observational cohort studies showed that low-serum albumin levels led to increased risk of VTE in the different populations (5, 6, 8). Metabolic syndrome (MS), characterized by changes in the metabolites including blood glucose, high-density lipoprotein cholesterol (HDL-C), and triglycerides levels, has been identified as a risk factor for various cardiovascular diseases including VTE in several studies (9). Besides, several publications have demonstrated that the use of lipid-lowering drugs decreases the risk of VTE (10–12). Hyperuricemia, hyper-homocysteinemia, and other metabolic dysregulations are also found in the patients with MS (13). However, the causal associations of the circulating metabolites with the risk of VTE are still unclear as conflicting results exist (7). Also, the evidence provided by the previous observational studies may be compromised by confounding factors or reverse causality. Mendelian randomization (MR), which is an epidemiological method exploiting genetic variants associated with exposures for appraising and strengthening causal inferences, diminishes the effect of confounding factors and reverse the causality.

Recent genome-wide association studies (GWASs) have identified the underlying genetic determinants of different circulating metabolites (14, 15). The MR method has been used by several studies for assessing the causal associations between low-serum albumin levels and the increased risk of heart failure and atrial fibrillation (16, 17). Genetic instruments associated with the lipid profile have also been used to examine the effects of lipid levels on several diseases (18, 19). Recent MR studies suggested that the several risk factors are causally associated with the risk of VTE, including taller height, obesity, and red blood cell traits (20–22). However, evidence about the causal associations between circulating metabolites and VTE risk are still lacking. Here, we performed two-sample MR analyses to investigate the relationships between several circulating metabolites [serum albumin, total protein levels, lipid profiles, branched-chain amino acids (BCAAs), etc.] and VTE.

## Materials and Methods

### Study Design

**Figure 1:**
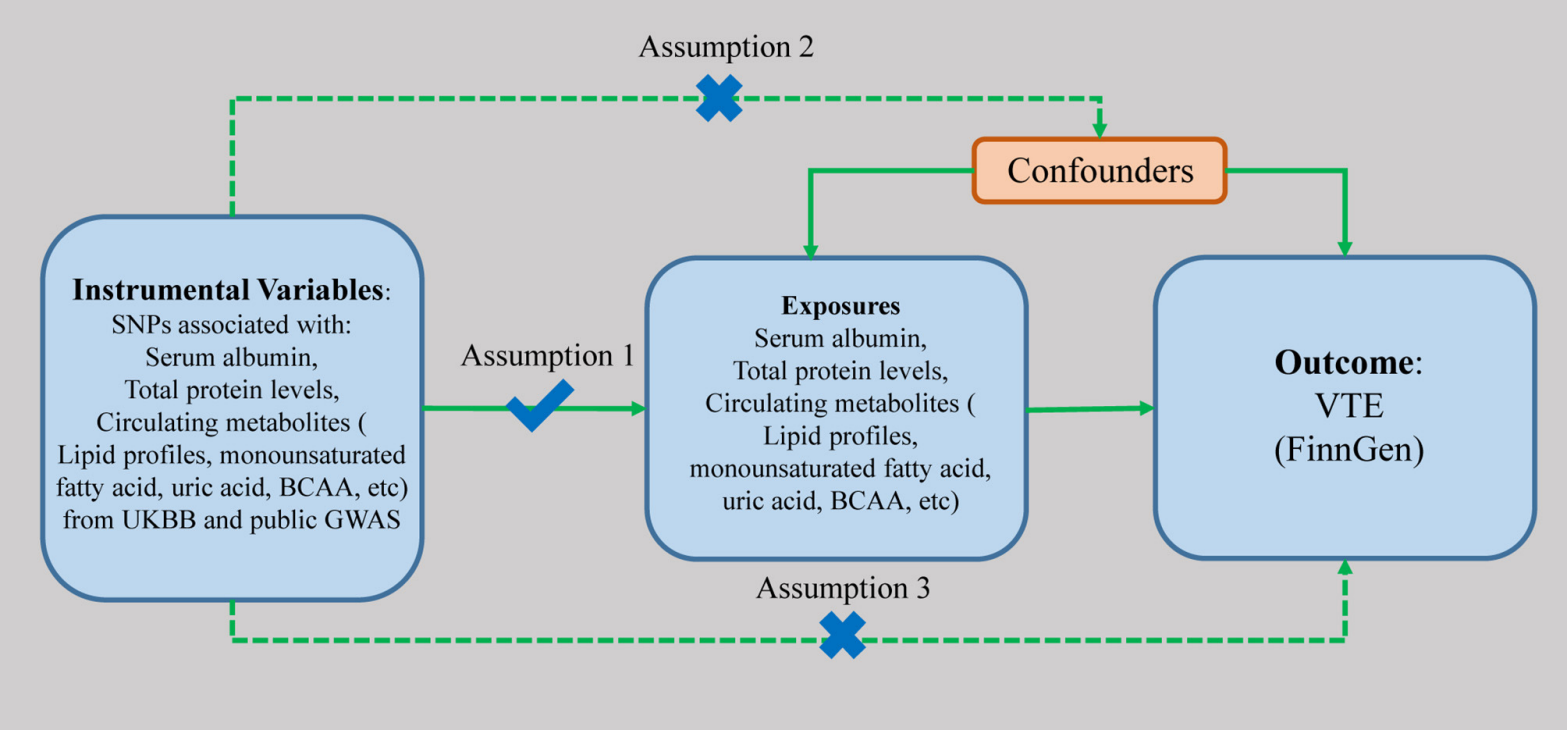
The scheme of study design. SNP, single nucleotide polymorphism; UKBB, UK BioBank; BCAA, branched-chain amino acid; GWAS, genome-wide association study; VTE, venous thromboembolism.

The schematic view of the study design and three assumptions of MR are presented in Figure 1. The genetic variants associated with the serum albumin and various circulating metabolites were used as genetic instruments (IVs) to examine the causal associations with the risk of VTE. There are three major assumptions that need to be satisfied to perform the MR analyses. First, these genetic variants are supposed to have a direct effect on VTE. Second, the genetic variants have no associations with any known confounding factors. At last, the effects of the genetic variants on VTE should only be mediated by the exposures. Genetic instruments associated with exposures are extracted from previously published GWASs (Table 1). The genetic associations between instrumental variables and the traits were all adjusted for age, sex, and study-specific covariates in the included GWASs. All the included studies had obtained ethical approvements from a relevant institutional review board, and all the subjects included had provided signed informed consent.

**Table 1:** Information of the data sources for exposures included in this Mendelian randomization study.

**Exposures**	**Sample size**	**Year**	**SNP number**	**PMID**	**Authors**	***F*-statistic**	**ID**
Serum albumin	315,268	2018	269	NA	NA	73.37	ukb-d-30600_irnt
Total protein	314,921	2018	346	NA	NA	66.96	ukb-d-30860_irnt
HDL-cholesterol	187,167	2013	124	24097068	Willer et.al	119.91	ieu-a-299
LDL-cholesterol	173,082	2013	100	24097068	Willer et.al	154.17	ieu-a-300
Total cholesterol	187,365	2013	118	24097068	Willer et.al	127.20	ieu-a-301
Triglycerides	177,861	2013	70	24097068	Willer et.al	150.24	ieu-a-302
Monounsaturated fatty acids	115,078	2020	83	NA	NA	113.31	met-d-MUFA
Saturated fatty acids	115,078	2020	69	NA	NA	104.38	met-d-SFA
Ratio of monounsaturated fatty acids to total fatty acids	115,078	2020	86	NA	NA	129.12	met-d-MUFA_pct
Omega-6 fatty acids	115,078	2020	70	NA	NA	106.04	met-d-Omega_6
Omega-3 fatty acids	115,078	2020	76	NA	NA	148.23	met-d-Omega_3
Apolipoprotein B	439,214	2020	325	32203549	Richardson et.al	170.24	ieu-b-108
Apolipoprotein A-I	393,193	2020	492	32203549	Richardson et.al	122.74	ieu-b-107
Lipoprotein(a)	273,896	2018	51	NA	NA		ukb-d-30790_irnt
Valine	115,078	2020	23	NA	NA	74.75	met-d-Val
Leucine	115,078	2020	16	NA	NA	64.53	met-d-Leu
Isoleucine	115,078	2020	7	NA	NA	79.89	met-d-Ile
Total concentration of branched-chain amino acids (leucine + isoleucine + valine)	115,078	2020	16	NA	NA	75.41	met-d-Total_BCAA
HbA1C	46,368	2010	11	20858683	Soranzo et.al	77.61	ieu-b-103
3-Hydroxybutyrate	115,078	2020	16	NA	NA	64.08	met-d-bOHbutyrate
Uric acid	109,029	2019	75	29403010	Kanai et.al	160.29	bbj-a-57
Homocysteine	44,147	2013	14	23824729	Meurs et.al	84.71	GCST002087

### Data Source and SNP Selection

Summary level data of the serum albumin and total protein levels were obtained from the UK BioBank cohort (https://www.ukbiobank.ac.uk/). Summary level data of HDL, low-density lipoproteins (LDL), total cholesterol, and triglycerides levels were obtained from the Global Lipids Genetics Consortium (GLGC) (15). Detailed information of data source for the instrumental variables associated with other exposures is presented in Table 1. Instrumental strength has been characterized by mean *F*-statistic with the approximation method described by Bowden et al. to test for weak instruments (23) (Table 1, Supplementary Table 2).

Single-nucleotide polymorphisms (SNPs) were identified as associated with the exposures with *p*-values at the genome-wide significance level (*p* < 5 × 10–8). SNPs with *R*^2^ > 0.01 and within 5,000 kb distance were identified as in linkage disequilibrium and were excluded from the study. For serum albumin and total protein levels, 111 and 146 SNPs were extracted and used as instrumental variables, respectively. The number of used SNPs and details of included SNPs are shown in Table 1 and Supplementary Table 2. Associations of these SNPs with VTE were studied in summary level results including 6,913 patients and 1,69,986 controls from the FinnGen consortium (release 4, https://r4.finngen.fi/).

### Statistical Methods

The inverse-variance weighted (IVW) method was used as the main method to assess the causal associations between the exposures and VTE, supported by the multiple sensitivity analyses including MR-Egger, weighted median, and MR-PRESSO (Mendelian Randomization Pleiotropy RESidual Sum and Outlier) methods. MR-Egger method can identify potential pleiotropy (*p* for intercept < 0.05) and give corrected estimates (24). MR-PRESSO method was used for detecting and correcting for the potential outliers (25). The weighted median method can give consistent causal estimates even when half the weight in the MR analysis came from invalid instrumental variables (26). We used leave-one-out analysis to evaluate the stability of these genetic variants by excluding one individual SNP each time.

All statistical results are two-sided, and a *p* < 0.0022 (0.05/23 adjusted with Bonferroni method) was considered statistically significant, *p* between 0.05 and 0.0022 were considered suggestive significant. The statistical analyses were performed with *R* (version 4.0.2), TwoSampleMR (0.5.5), MR (0.5.0), and MR-PRESSO packages (27, 28).

## Results

**Figure 2:**
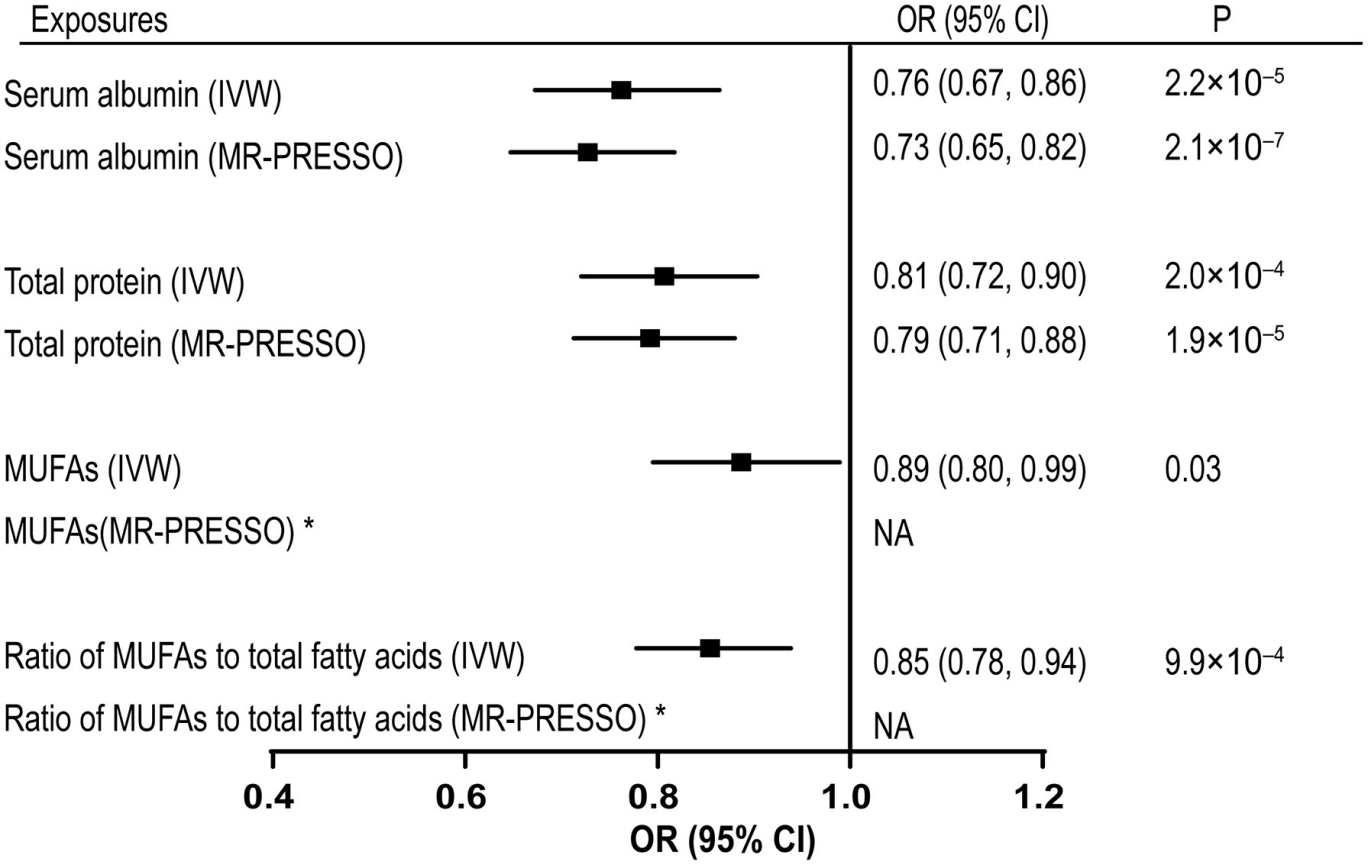
Associations of genetically predicted serum albumin, total protein, MUFAs levels, and ratio of MUFAs to total fatty acids with VTE. MUFAs, monounsaturated fatty acids; VTE, venous thromboembolism; IVW, inverse-variance weighted; MR-PRESSO, Mendelian Randomization Pleiotropy RESidual Sum and Outlier; OR, odds ratio; CI, confidence interval. *No outliers detected. NA, no adjusted estimates is available.

The associations with VTE per one SD increase in the genetically predicted serum albumin and total protein levels are shown in Figure 2. Genetically proxied serum albumin and total protein levels were inversely correlated to VTE, with odds ratios (ORs) equal to 0.69 (CI 0.54–0.89, *p* = 4.7 × 10^−3^) and 0.76 (CI 0.61–0.95, *p* = 0.015) per one SD increase by using IVW method. MR-PRESSO detected no outliers in the analyses and gave causal estimates consistent with results from IVW (Serum albumin: OR 0.83, 95% CI 0.73–0.94, *p* = 5.6 × 10^−3^; Total protein: OR 0.87, 95% CI 0.78–0.97, *p* = 0.016). No horizontal pleiotropy was identified with MR-egger method (Serum albumin: *p* = 0.23; Total protein: *p* = 0.66). We further replicated the MR analyses with VTE data from UK BioBank. Associations of both the serum albumin and total protein levels with VTE remained in the same direction and reached *p* < 0.05 (data not shown).

Genetically proxied monounsaturated fatty acids (MUFAs) and the ratio of monounsaturated fatty acids to total fatty acids were causally associated with the risk of VTE by using the IVW method, with ORs of 0.89 (CI 0.80–0.99, *p* = 0.031) and 0.85 (CI 0.78–0.94, *p* = 9.92 × 10^−4^), respectively (Supplementary Table 1). No outliers were detected in the SNPs associated with these two exposures. However, the MR-egger method detected significant pleiotropic effects on the SNPs used for the ratio of monounsaturated fatty acids to total fatty acids, indicating potential confounding effect (intercept = 0.010, *p* = 0.028). The estimates provided by MR-egger supported a causal association between the ratio of monounsaturated fatty acids to total fatty acids and VTE risk (OR = 0.75, CI 0.65–0.87, *p* = 2.19 × 10^−4^) (Supplementary Table 1). No causal association was identified between other circulating metabolites and the risk of VTE (Supplementary Table 1). Scatter plots for the associations of included 22 exposures with VTE are shown in Supplementary Figures 1–22. Leave-one-out plots (serum albumin, total protein, MUFAs, ratio of MUFAs to the total fatty acids) were shown in Supplementary Figures 23–26.

## Discussion

This MR study demonstrated that the genetically proxied serum albumin, total protein levels, MUFAs, and the ratio of MUFAs to the total fatty acids are inversely associated with the risk of VTE. There is no indication of causal effects of other circulating metabolites, including the measurements in the lipid profiles, BCAA, glucose, uric acid, homocysteine, carnitine, and three-hydroxybutyrate on the risk of VTE.

### Serum Albumin and Total Protein With VTE

Several observational cohort studies indicated that a lower level of serum albumin level can increase the risk of VTE in the different individuals (8, 29). The study based on the two population cohorts: the Atherosclerosis Risk in Communities (ARIC) Study and the Cardiovascular Health Study (CHS) suggested that the low-serum albumin level is the modest marker of increased risk of VTE with the adjusted hazard ratio (HR) per SD lower albumin equal to 1.18 (95% CI = 1.08, 1.31) in the ARIC and 1.10 (95% CI = 0.94, 1.29) in CHS, after adjustment with other VTE-associated risk factors (29). Another long-term cohort, which enrolled 2,176 male individuals in the Caucasian ancestries with an average follow-up of 24.8 years, also showed that one SD lower serum albumin was associated with a higher risk of VTE, with HR = 1.23 (95% CI 1.02–1.47), and this association was independent of CRP levels (8). In addition, the cancer patients and individuals with nephrotic syndromes were also shown to be more likely to develop VTE (5). Our study, for the first time, by using MR method, demonstrated that the higher levels of serum albumin and total protein levels show a causal protective effect on the risk of VTE based on GWASs from the relatively large populations. Of note, our results are less susceptible to biases including the confounders and reverse causality. This is of particular importance in disease conditions considering the dramatic changes in circulating metabolites in the human microenvironment. Therefore, our data put forward another layer of evidence supporting the causal associations of the serum albumin and total protein levels with the risk of VTE.

### Metabolic Syndrome-Associated Metabolites With VTE

#### Lipid Profiles and VTE

Previous observational studies provided conflicting evidence about the associations of several traits in lipid profiles and the risk factors of VTE. For example, one study demonstrated that Lp(a) levels above 30 mg/dl were more than twice likely to be found in the patients with VTE as compared with the controls (30). An observational cohort study enrolling 27,081 healthy women with an average follow-up of 11.4 years showed no causal relationships between any lipid profile trait and VTE. Subgroup analyses in individuals taking hormonal replacement therapy suggested that extreme tertile of HDL-C and apo-AI levels were associated with an increased risk of unprovoked VTE (9). However, our MR analyses showed no causal associations between any of the lipids with VTE. The conflicting results might be because of the biases led by limited sample size or uncharacterized confounding factors in the observational studies. Of note, our results are supported by the Prevention of Renal and Vascular Endstage Disease (PREVEND) study, which is based on a large cohort including all the habitant aged 28–75 years living in Groningen, the Netherland (31). The PREVEND study showed that the apolipoproteins were not associated with a higher risk of VTE in both the univariable and multivariable analyses. Although the classical lipoproteins: TC, non-HDL, LDL, TG, and TC/HDL ratio were showed to increase the risk of VTE in the univariable analyses, the significance was absent after the multivariable adjustment with age and sex (31).

Likewise, we did not see causation of the saturated fatty acid, omega-3 fatty acid, and omega-6 fatty acid with VTE, though multiple previous studies suggested a protective effect of long-chained *n*-3 polyunsaturated fatty acids (*n*-3 PUFAs) with the risk of VTE (32–35). This might be because these studies were focusing on hospitalized patients, therefore confounding factors such as immobilization and liver function deficiency might play additional roles in promoting VTE. However, we saw a suggestive protective effect of MUFAs and the ratio of MUFAs to total fatty acid level. The roles of MUFAs in inhibiting pro-inflammatory gene expression and anti-oxidation have been widely studied (36). Further studies are required to investigate whether the anti-inflammatory effects from MUFAs can exert a beneficial effect on preventing VTE. Our study is a complement to a recent MR analysis, showing specific protective effect of MUFA on the risk of VTE without affecting the risk of other major cardiovascular disorders (37).

#### HbA1C, Uric Acid, Homocysteine, and VTE

Our MR study also suggested that the genetically proxied HbA1C is not associated with high-VTE risk. These data are in line with previous findings from the population-based cohorts (38–40). Venous thromboembolism In Northern Sweden (VEINS) cohort study suggested that not only fasting plasma glucose (FPG), oral glucose tolerance test [2-h post-load plasma glucose (2HPG)] but clinically also diagnosed diabetes have no association with increased risk of VTE after multiple confounder adjustment (age, sex, body mass index, cancer at inclusion, education level, smoking, and hypertension) (39). The Tromsø study obtained similar results suggesting no relationships between high HbA1C levels and increased risk of VTE (38). Besides, though diabetes can increase the risk of VTE by using various models to adjust for the effects from confounders, elevated HbA1C levels in individuals without diabetes are not related to a higher risk of VTE (41).

Several cohort studies showed that serum uric acid (SUA) level is an independent risk factor of VTE (42–44). Though our MR data does not show a significant association between high level with VTE, the *p*-value is around the border of suggestive significance (*p* = 0.078) and suggested a trend toward increasing VTE risk. Likewise, there is no indication of elevated homocysteine in increasing the VTE risk, which suggested that the association seen between homocysteine and VTE might be due to confounders (45).

#### BCAA, 3-Hydroxybutyrate, and Other Metabolites With VTE

A previous pilot study applied nuclear magnetic resonance spectroscopy (NMR) and mass spectrometry (MS) method to measure the levels of BCAA, 3-hydroxybutyrate in plasma, serum, thrombus, and vein wall in human samples and suggested higher levels of BCAA and 3- hydroxybutyrate in individuals with VTE (46). However, the causations cannot be inferred and the associations between these metabolites and VTE are still elusive. Our data confirmed that there is no causal association between the levels of BCAA and 3-hydroxybutyrate with VTE.

### Limitations

There are several limitations with this study. First, we assumed that the correlations of different exposures with VTE are linear in the MR analysis. Second, we are unable to perform population stratification. The conclusions might be compromised if there are differences in allele frequency in different populations. Third, the results are based on European ancestry. Future studies on mixed populations or other populations are needed to extend our conclusion.

## Conclusion

In Summary, our MR study demonstrated the causal effects of genetically proxied lower serum, total protein levels, MUFAs, and the ratio of MUFAs to total fatty acids on the risk of VTE. Targeting these factors might be a potential strategy to prevent VTE.

## Data Availability Statement

Publicly available datasets were analyzed in this study. This data can be found at: https://r4.finngen.fi/.

## Author Contributions

JM and ZL conceptualized and designed the study, analyzed the data, and wrote the manuscript. All authors read and approved the final manuscript.

## Conflict of Interest

The authors declare that the research was conducted in the absence of any commercial or financial relationships that could be construed as a potential conflict of interest.

## Publisher's Note

All claims expressed in this article are solely those of the authors and do not necessarily represent those of their affiliated organizations, or those of the publisher, the editors and the reviewers. Any product that may be evaluated in this article, or claim that may be made by its manufacturer, is not guaranteed or endorsed by the publisher.

## Supplementary Material

Supplementary Figures 1–22 Scatter plot shows individual causal estimates from each genetic variant associated with each exposure on the x-axis and VTE on the y-axis.

Supplementary Figures 23–26 Leave-one-out analysis of serum albumin, total protein, MUFAs, and ratios of MUFAs to the total fatty acid.

Supplementary Table 1 Associations of 22 exposures with venous thromboembolism (VTE) in Mendelian randomization analyses.

Supplementary Table 2 Single-nucleotide polymorphisms used for each exposure.
