# Supplementary material for: Serum Albumin and Circulating Metabolites and Risk of Venous Thromboembolism: A Two-Sample Mendelian Randomization Study
Source: Front Nutr. 2021 Nov 11;8:712600. doi: 10.3389/fnut.2021.712600 (PMC8631825; doi:10.3389/fnut.2021.712600)
Supplement: Supplementary Figures 1–22 — Scatter plot shows individual causal estimates from each genetic variant associated with each exposure on the x-axis and VTE on the y-axis. [file Data_Sheet_1.PDF]

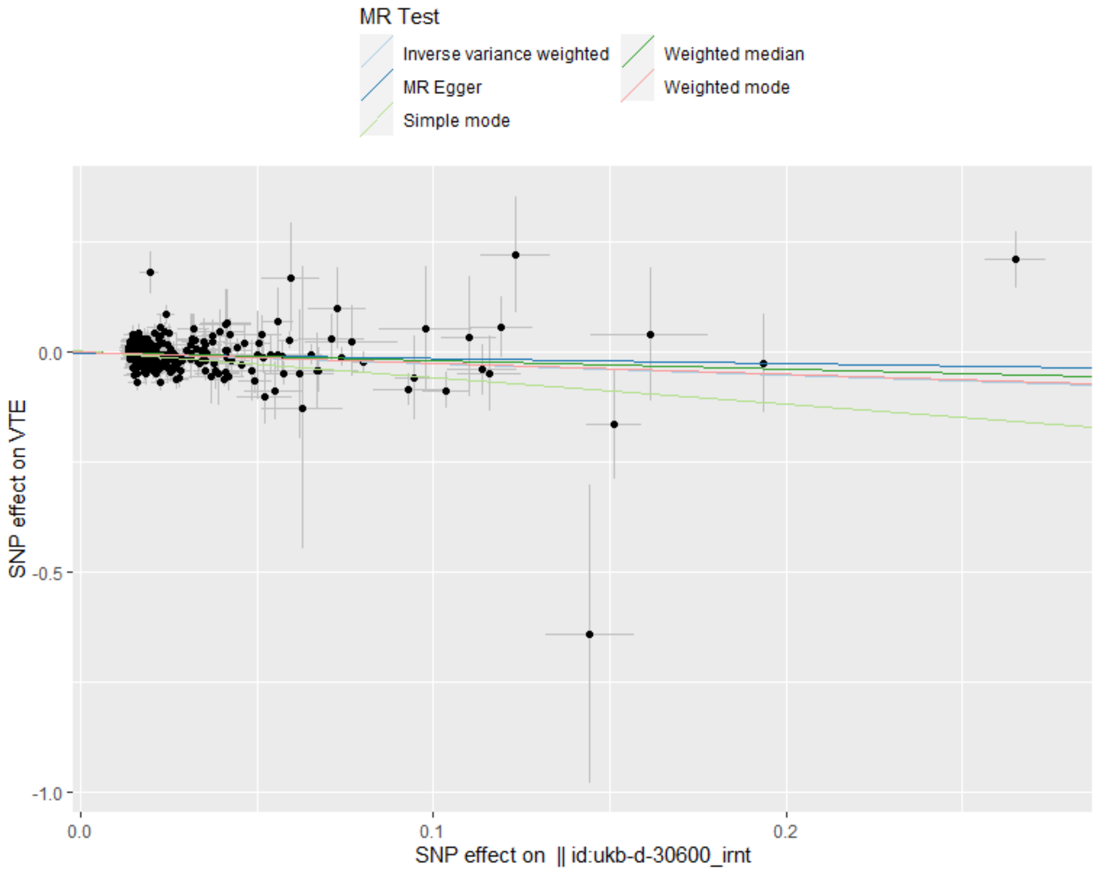

Supplementary figure 1: This scatter plot shows individual causal estimates from each genetic variant associated with serum albumin level on the x-axis and VTE risk on the y-axis.

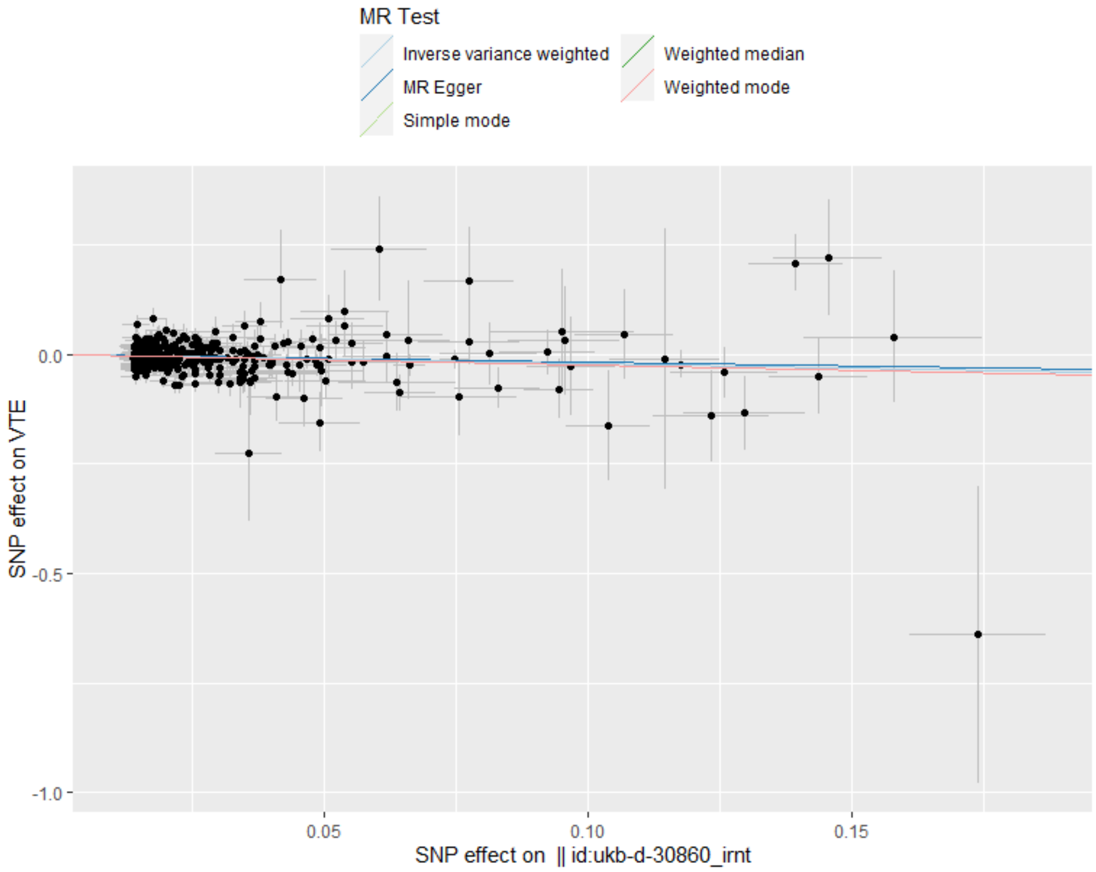

Supplementary figure 2: This scatter plot shows individual causal estimates from each genetic variant associated with total protein level on the x-axis and VTE risk on the y-axis.

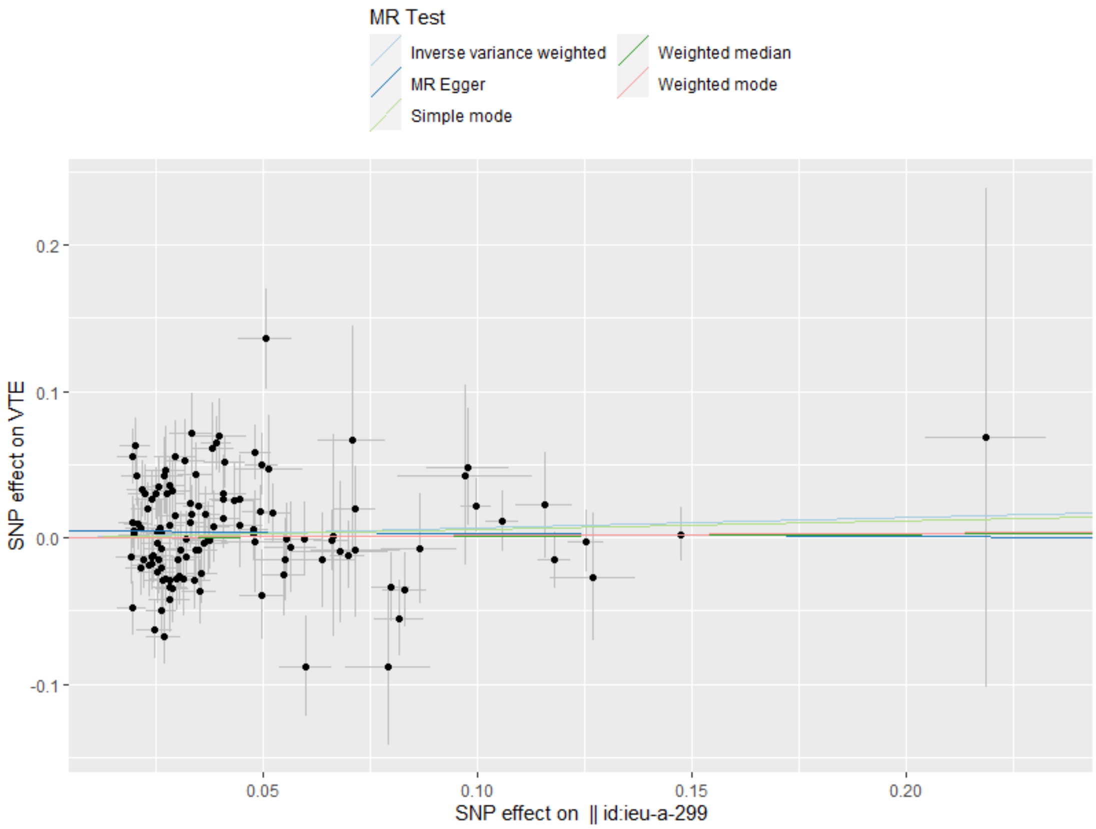

Supplementary figure 3: This scatter plot shows individual causal estimates from each genetic variant associated with HDL-C on the x-axis and VTE risk on the y-axis.

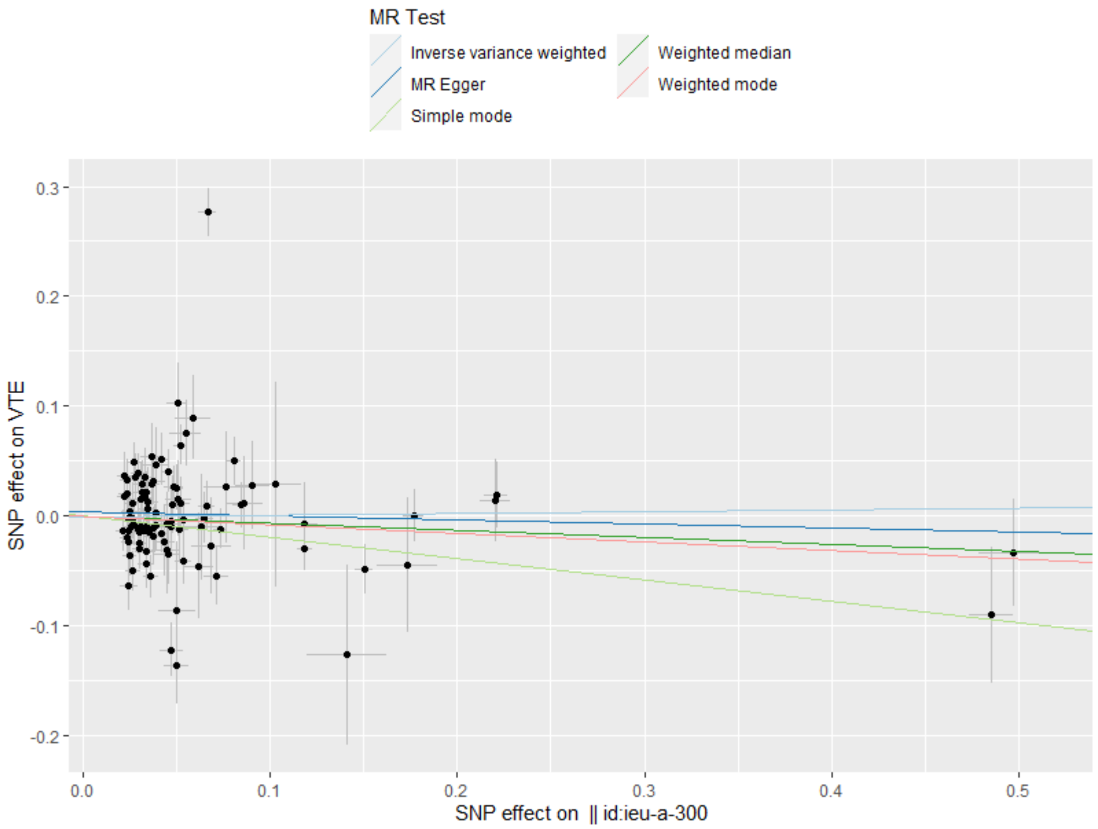

Supplementary figure 4: This scatter plot shows individual causal estimates from each genetic variant associated with LDL-C on the x-axis and VTE risk on the y-axis.

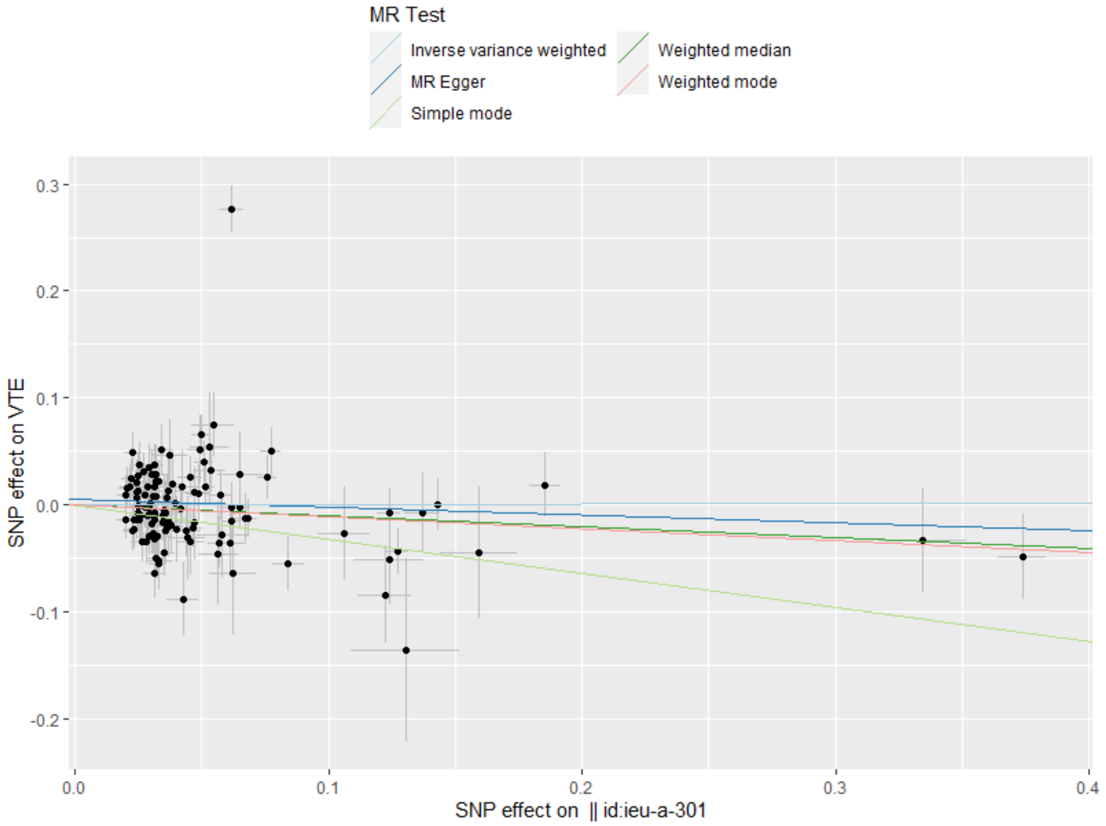

Supplementary figure 5: This scatter plot shows individual causal estimates from each genetic variant associated with total cholesterol on the x-axis and VTE risk on the y-axis.

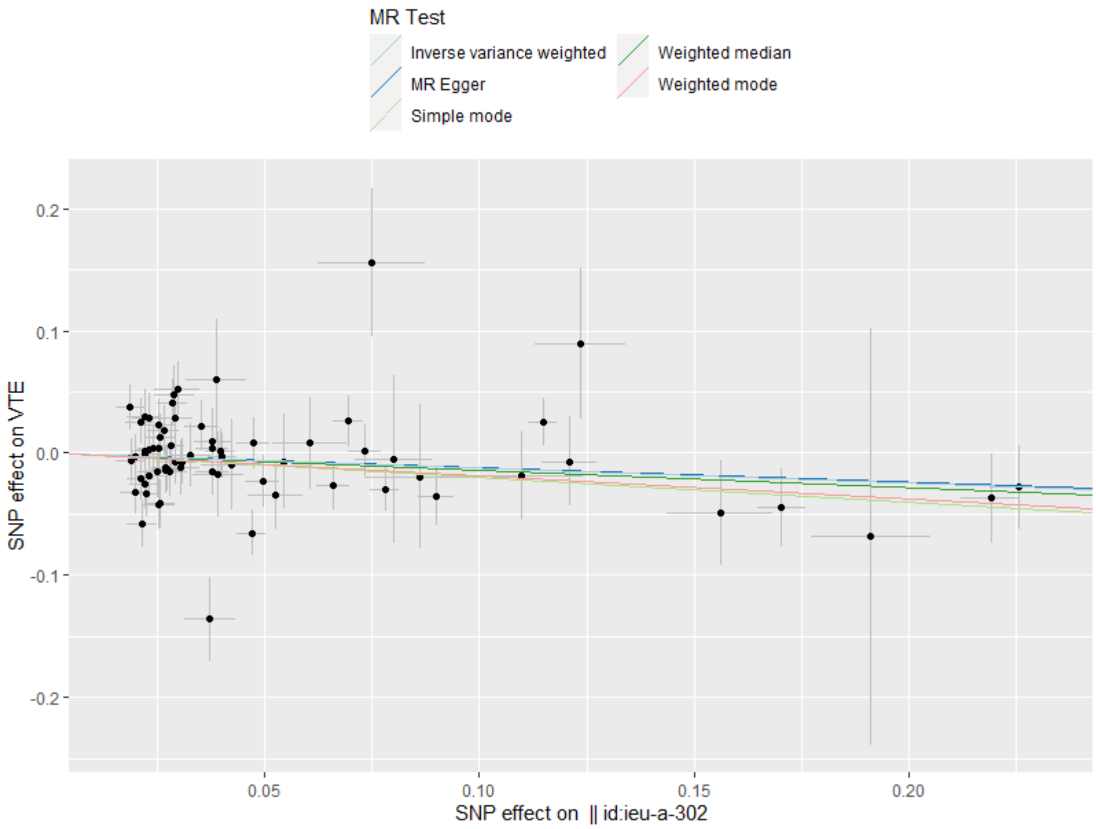

Supplementary figure 6: This scatter plot shows individual causal estimates from each genetic variant associated with triglycerides on the x-axis and VTE risk on the y-axis.

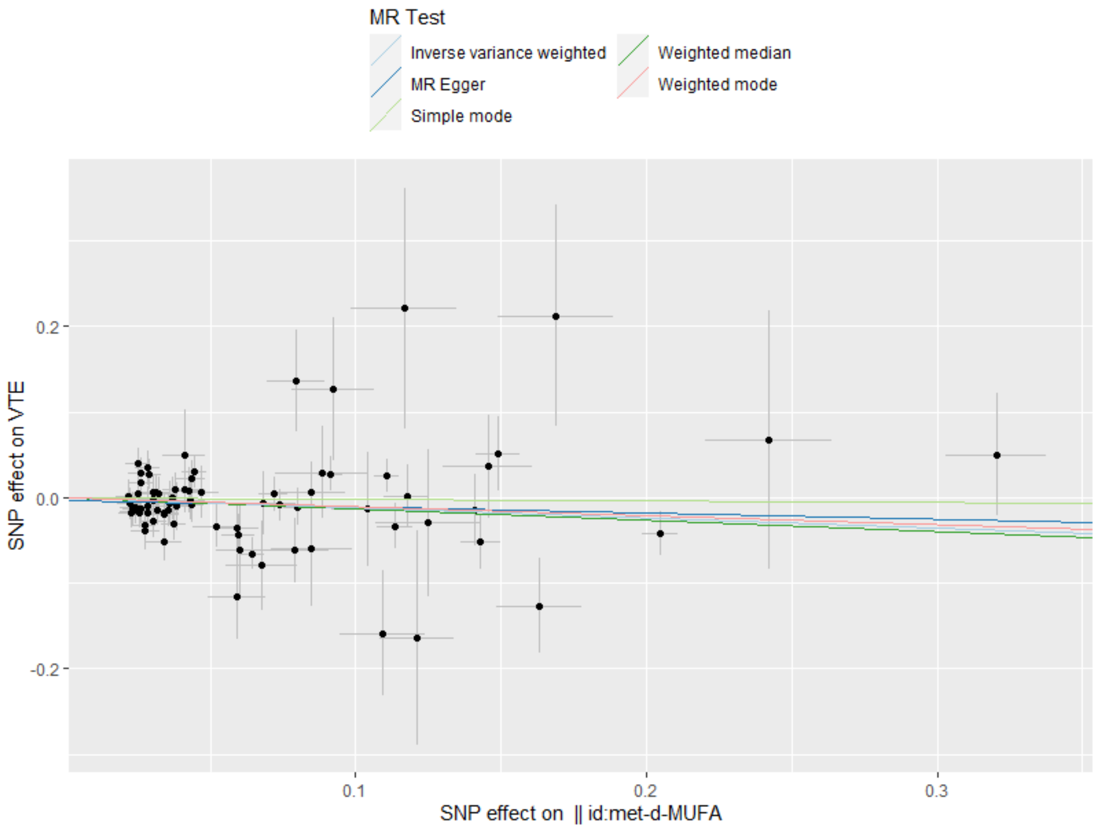

Supplementary figure 7: This scatter plot shows individual causal estimates from each genetic variant associated with monounsaturated fatty acid on the x-axis and VTE risk on the y-axis.

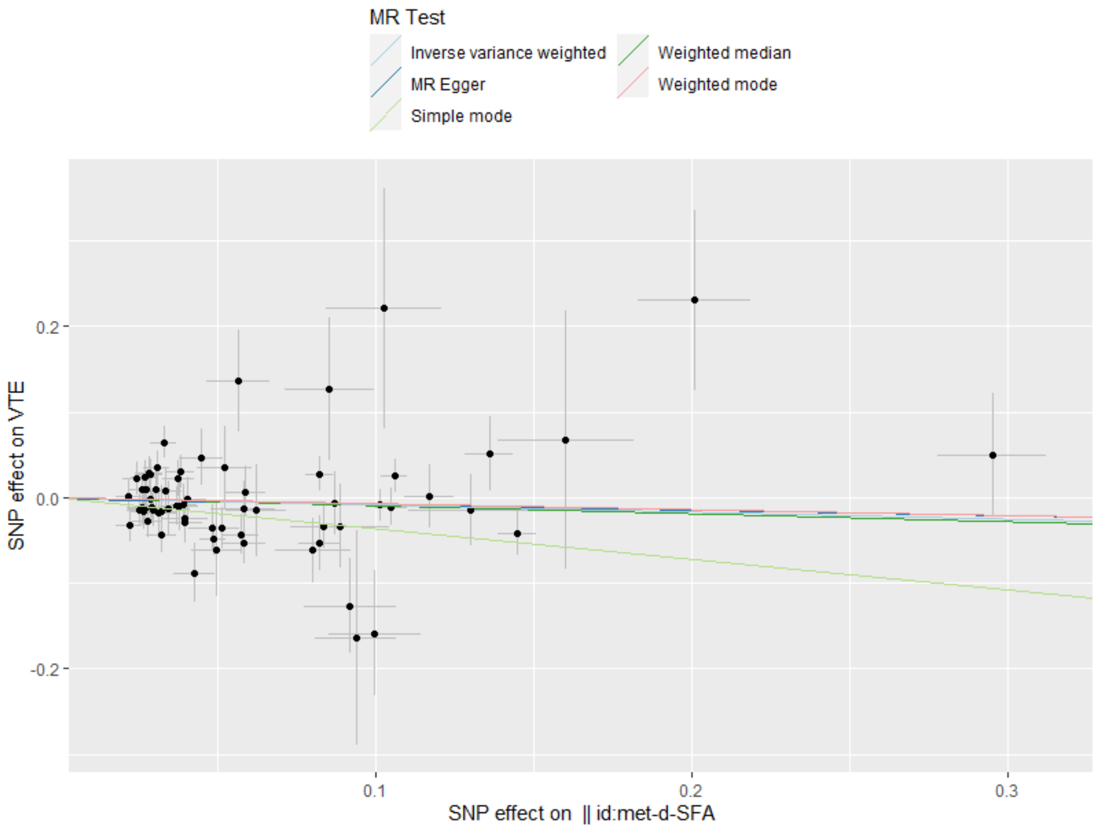

Supplementary figure 8: This scatter plot shows individual causal estimates from each genetic variant associated with saturated fatty acids on the x-axis and VTE risk on the y-axis.

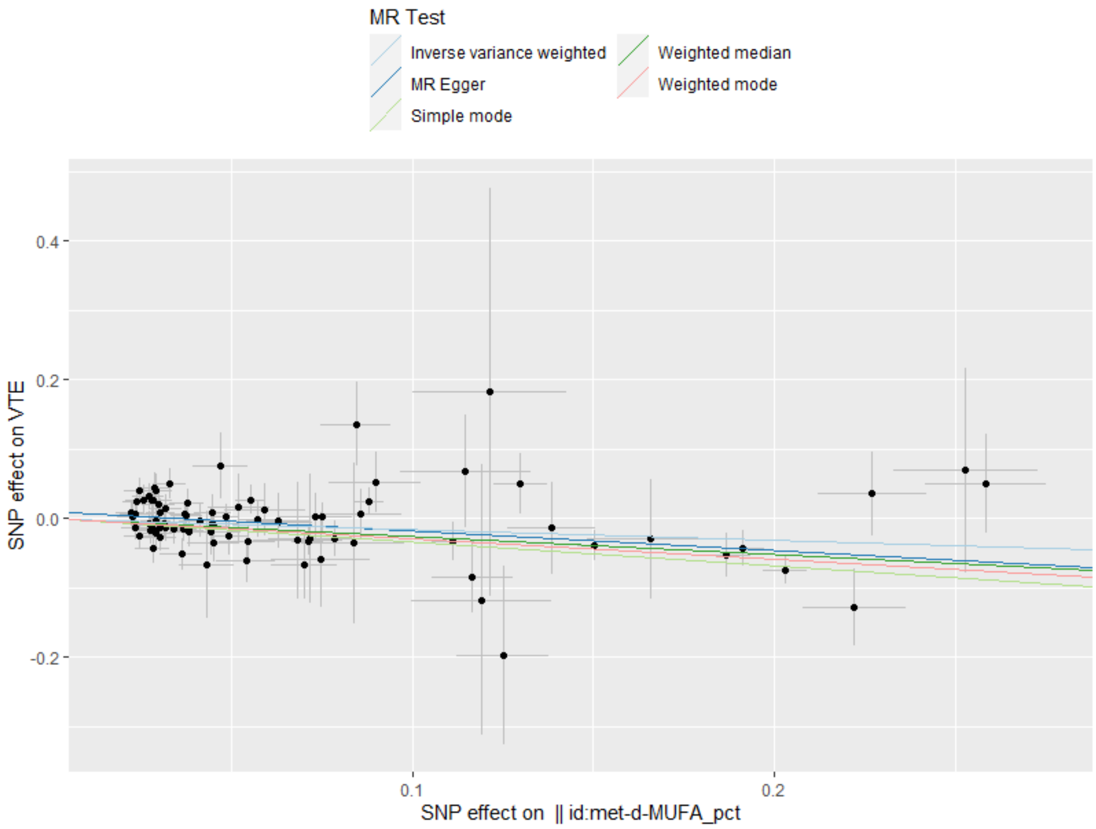

Supplementary figure 9: This scatter plot shows individual causal estimates from each genetic variant associated with the ratio of monounsaturated fatty acid to total fatty acids on the x-axis and VTE risk on the y-axis.

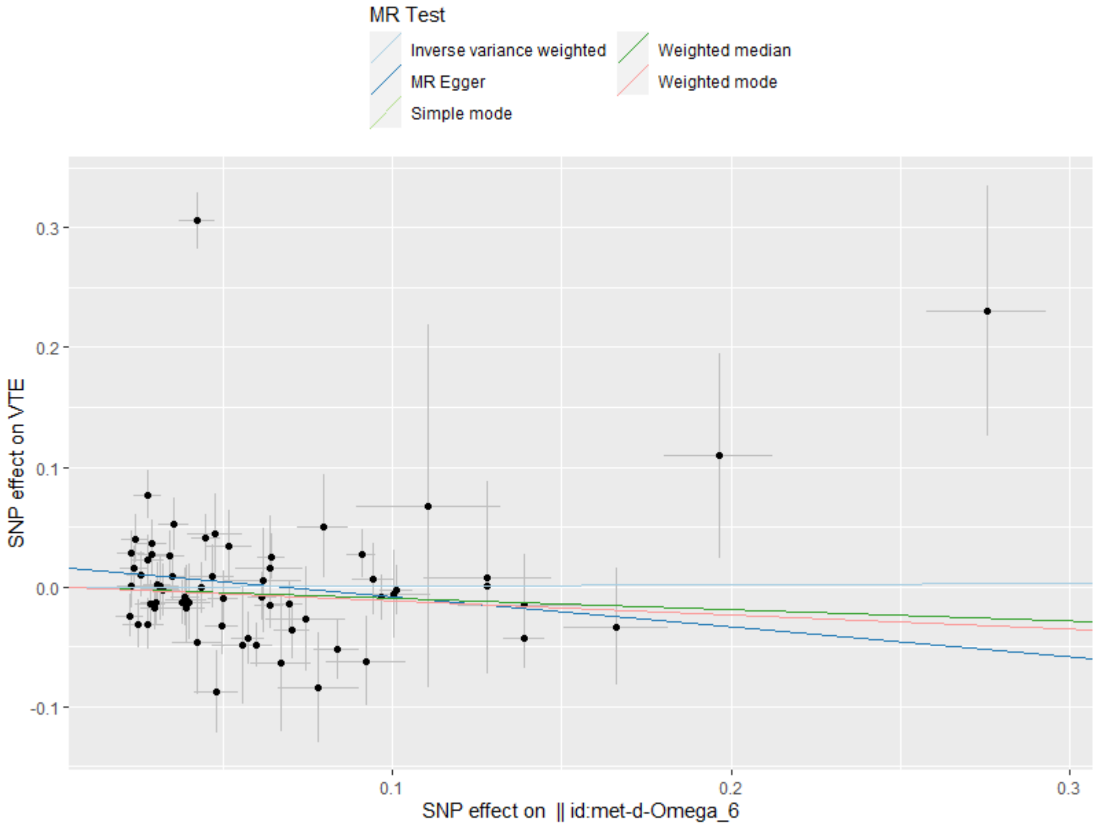

Supplementary figure 10: This scatter plot shows individual causal estimates from each genetic variant associated with Omega-6 fatty acids on the x-axis and VTE risk on the y-axis.

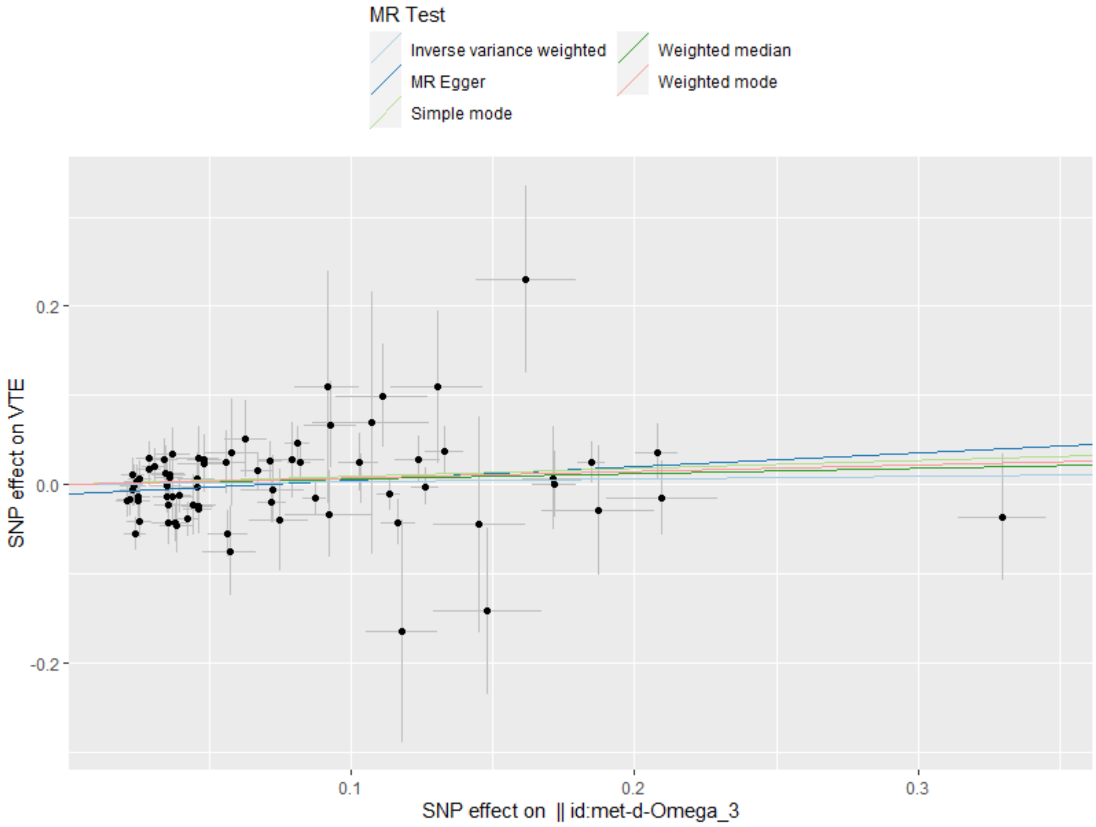

Supplementary figure 11: This scatter plot shows individual causal estimates from each genetic variant associated with Omega-3 fatty acids on the x-axis and VTE risk on the y-axis.

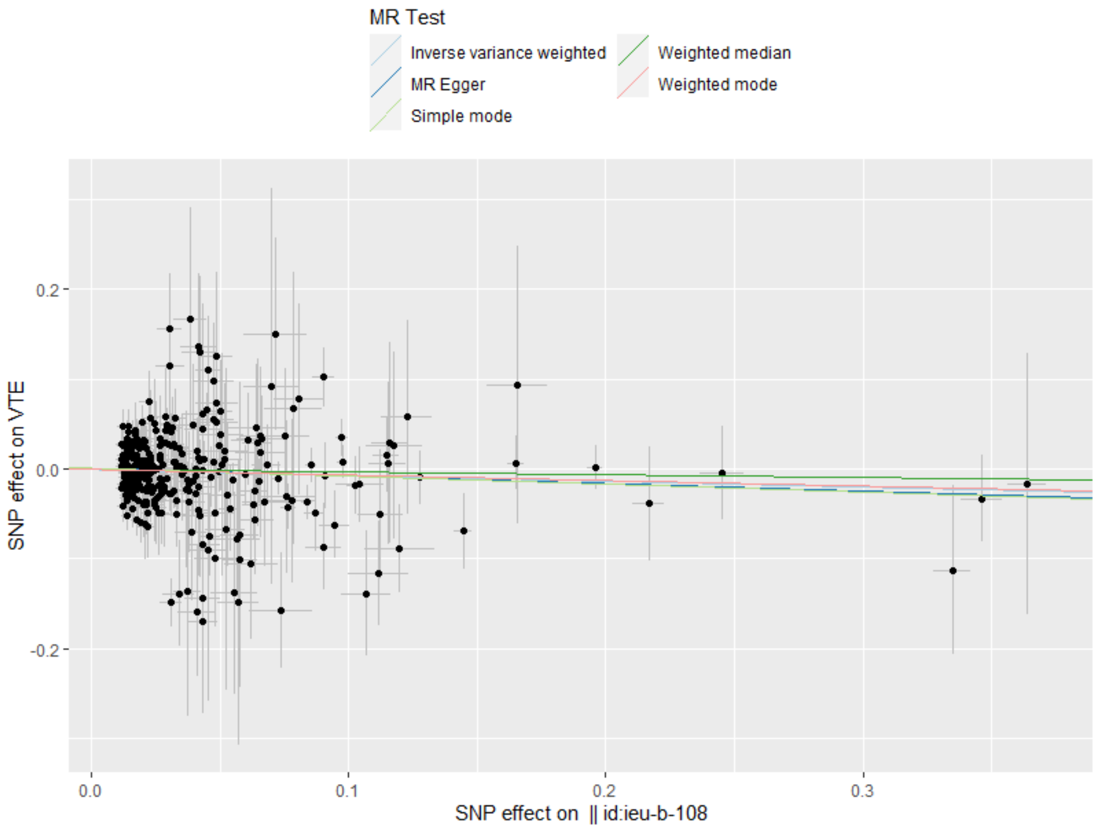

Supplementary figure 12: This scatter plot shows individual causal estimates from each genetic variant associated with Apolipoprotein B on the x-axis and VTE risk on the y-axis.

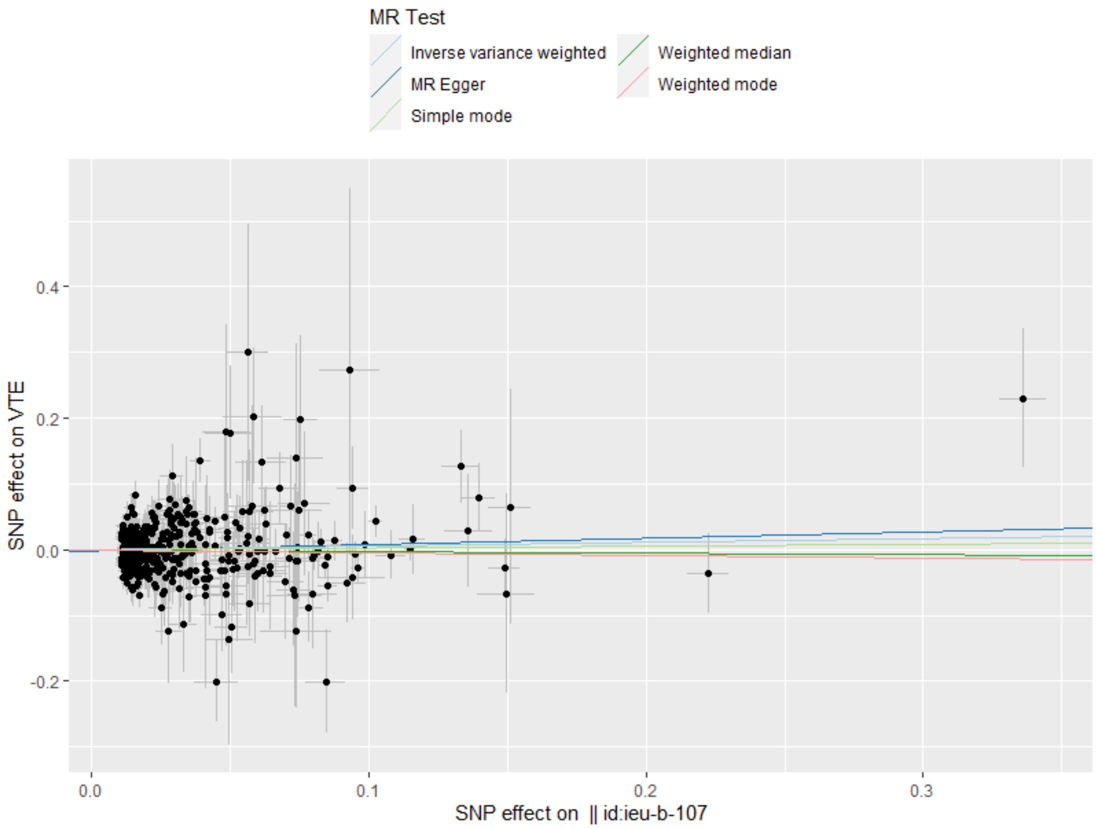

Supplementary figure 13: This scatter plot shows individual causal estimates from each genetic variant associated with Apolipoprotein A-I on the x-axis and VTE risk on the y-axis.

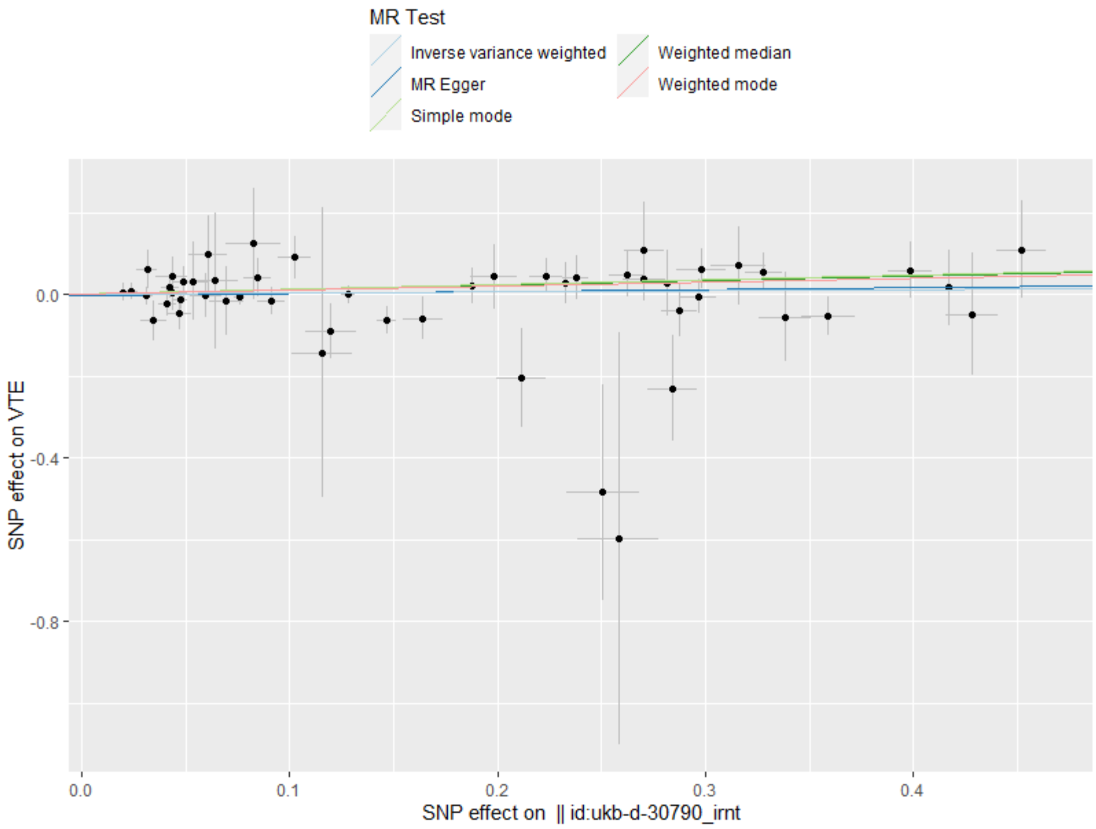

Supplementary figure 14: This scatter plot shows individual causal estimates from each genetic variant associated with Lp(a) on the x-axis and VTE risk on the y-axis.

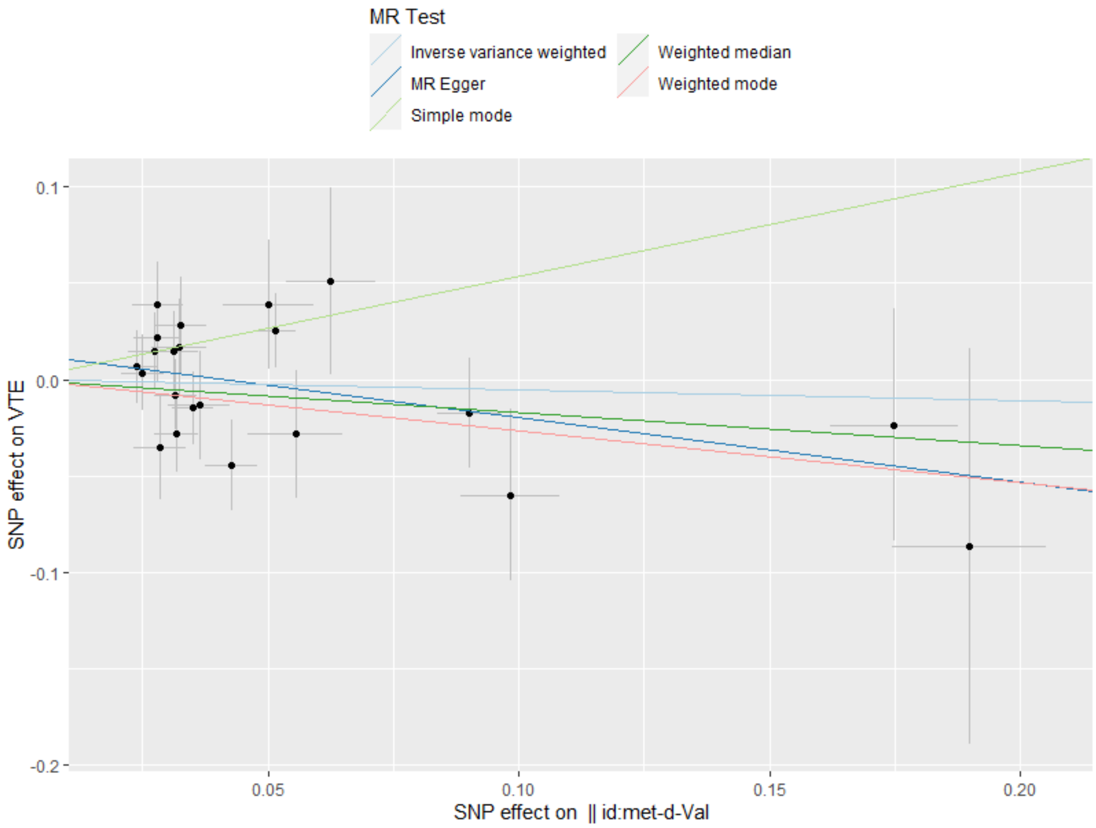

Supplementary figure 15: This scatter plot shows individual causal estimates from each genetic variant associated with valine on the x-axis and VTE risk on the y-axis.

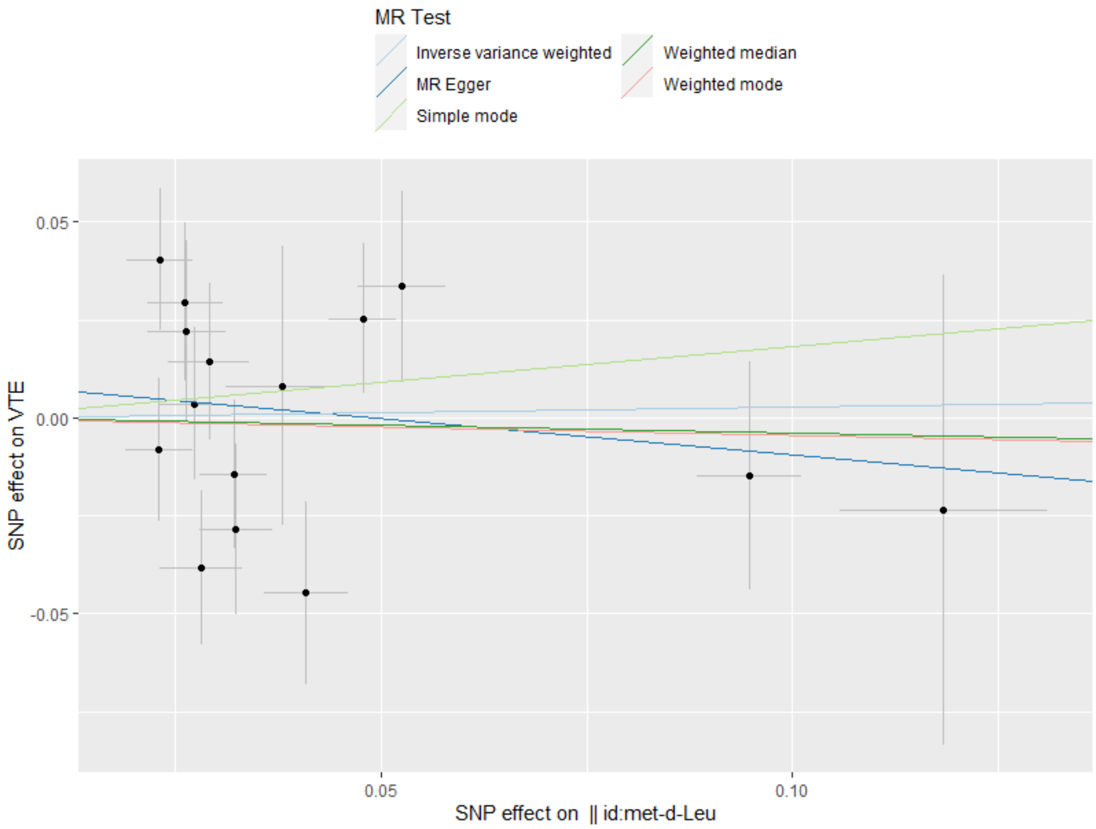

Supplementary figure 16: This scatter plot shows individual causal estimates from each genetic variant associated with leucine on the x-axis and VTE risk on the y-axis.

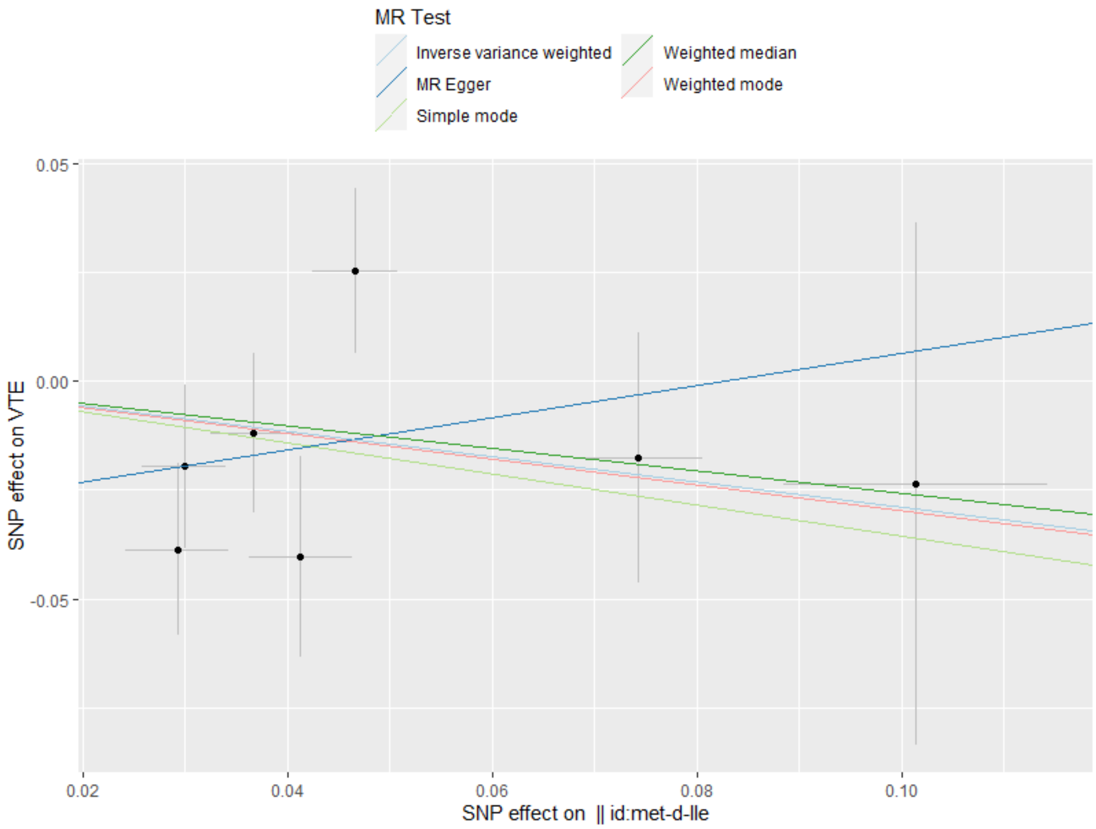

Supplementary figure 17: This scatter plot shows individual causal estimates from each genetic variant associated with isoleucine on the x-axis and VTE risk on the y-axis.

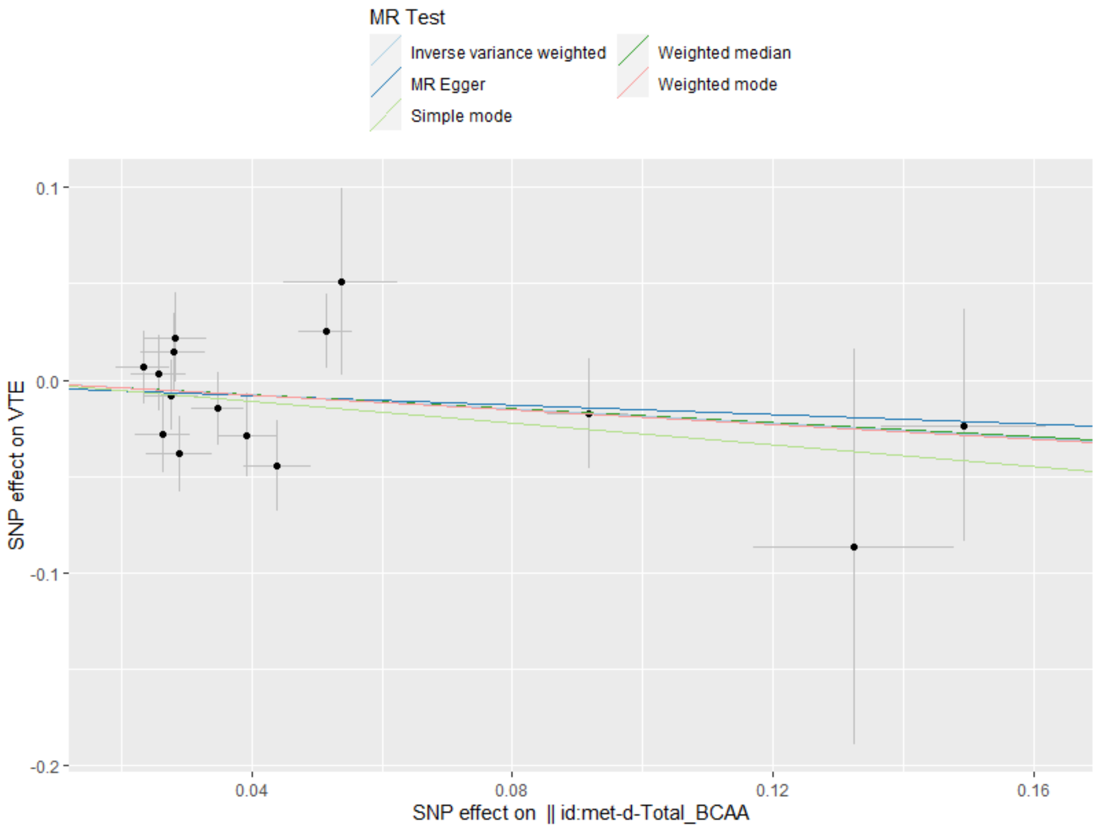

Supplementary figure 18: This scatter plot shows individual causal estimates from each genetic variant associated with the total concentration of BCAA on the x-axis and VTE risk on the y-axis.

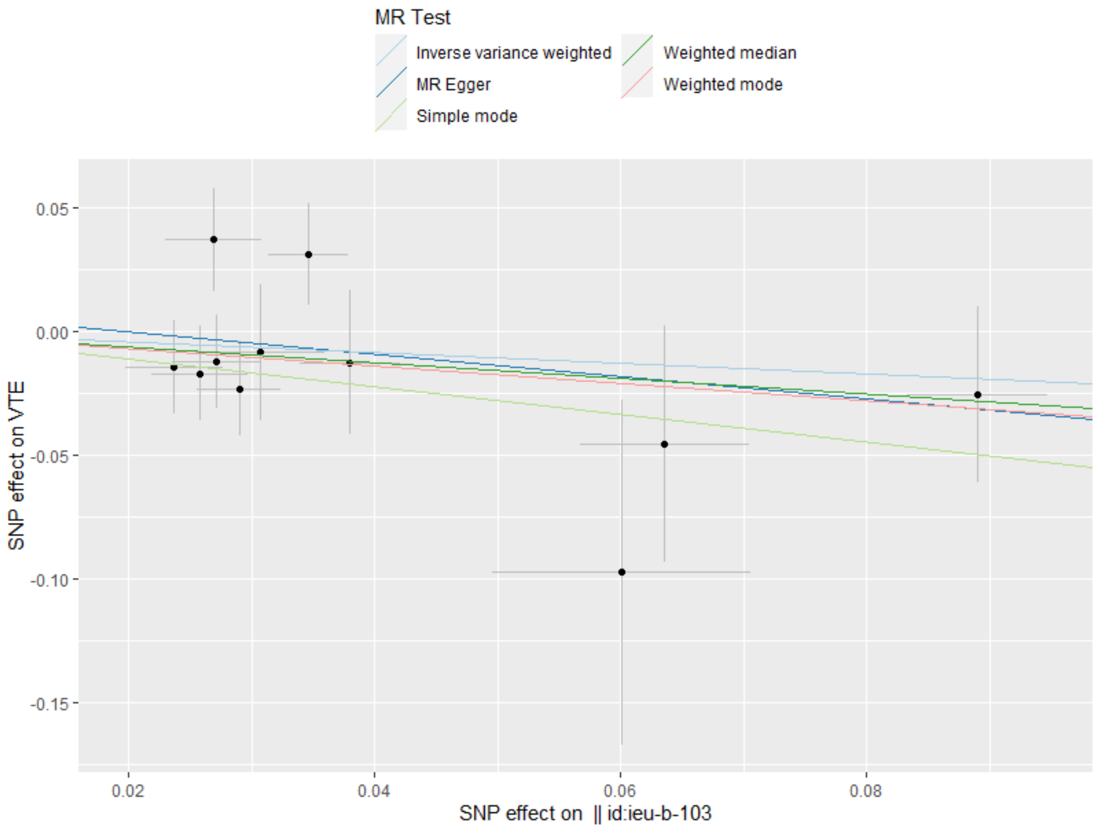

Supplementary figure 19: This scatter plot shows individual causal estimates from each genetic variant associated with HbA1C on the x-axis and VTE risk on the y-axis.

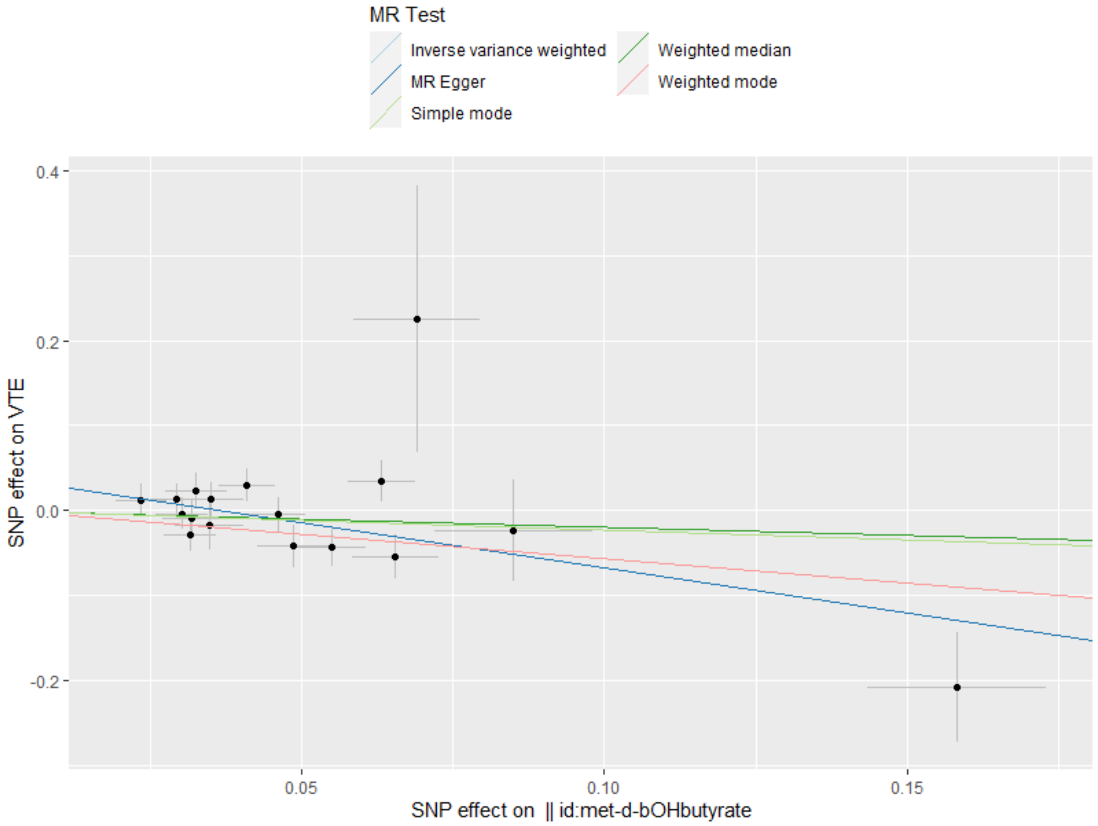

Supplementary figure 20: This scatter plot shows individual causal estimates from each genetic variant associated with 3-hydroxybutyrate on the x-axis and VTE risk on the y-axis.

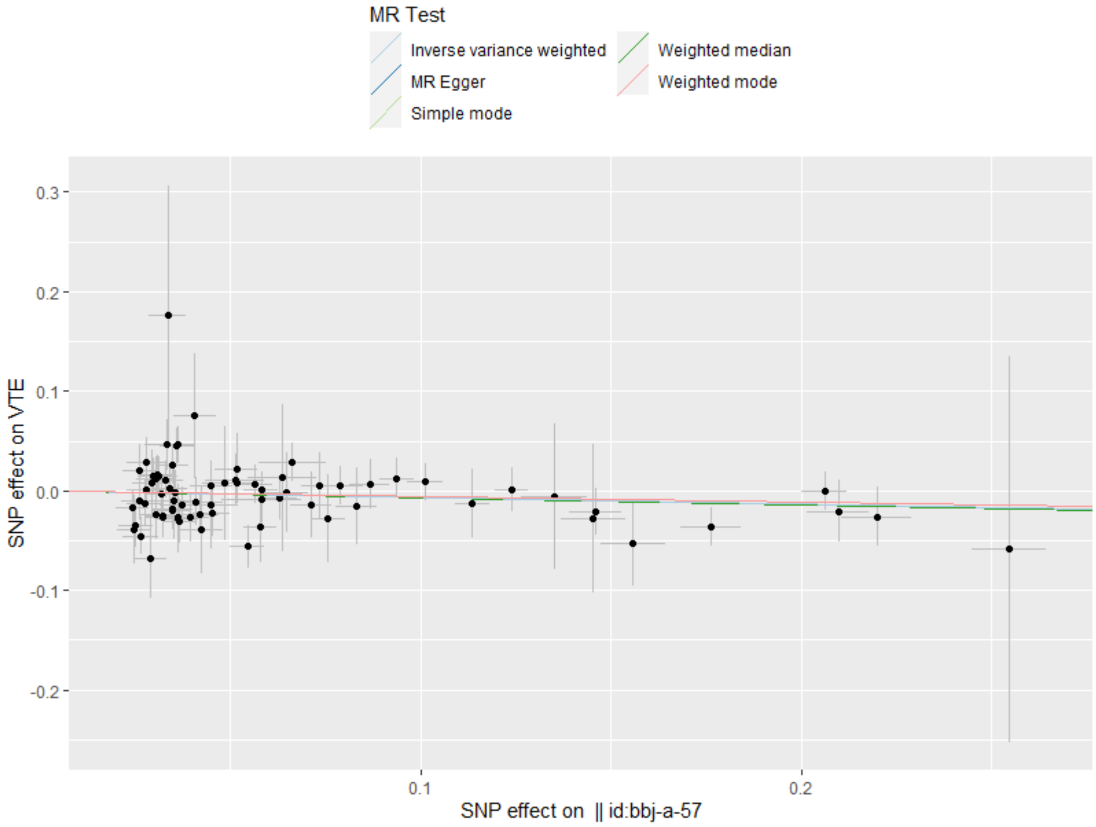

Supplementary figure 21: This scatter plot shows individual causal estimates from each genetic variant associated with uric acid on the x-axis and VTE risk on the y-axis.

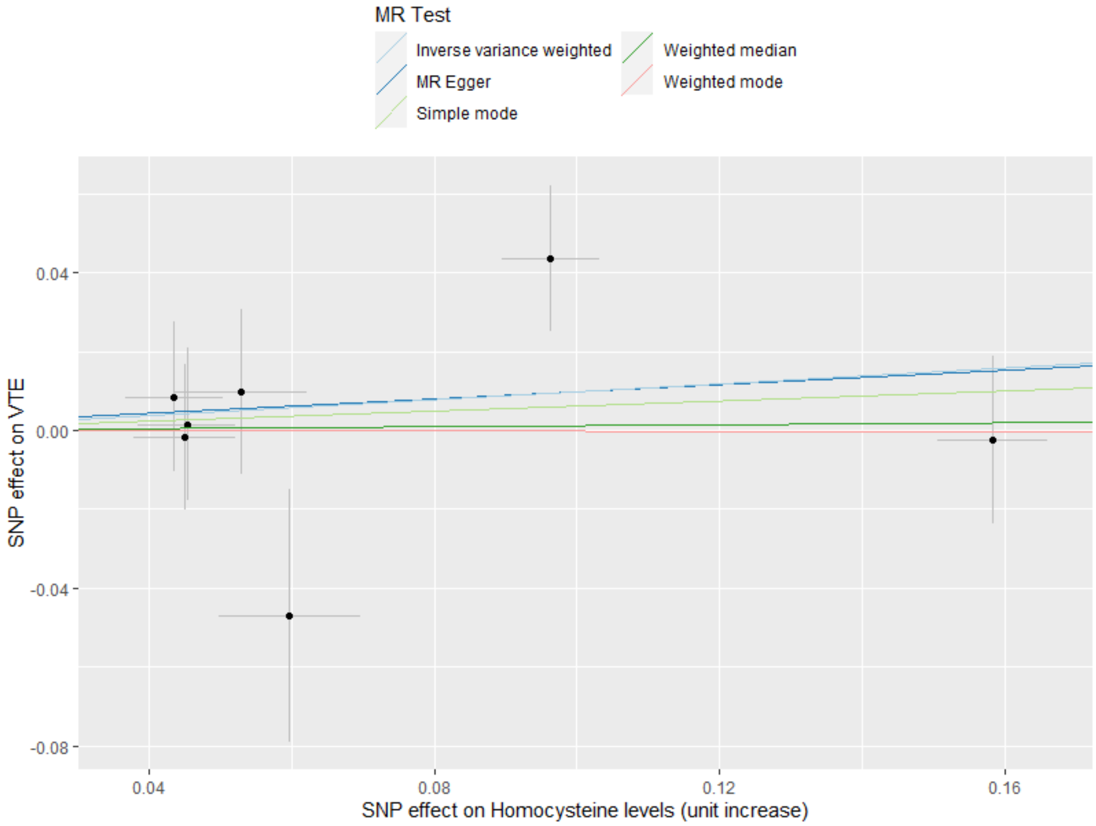

Supplementary figure 22: This scatter plot shows individual causal estimates from each genetic variant associated with homocystein on the x-axis and VTE risk on the y-axis.
