# Supplementary figures and images for: Serum Albumin and Circulating Metabolites and Risk of Venous Thromboembolism: A Two-Sample Mendelian Randomization Study
Source: Front Nutr. 2021 Nov 11;8:712600. doi: 10.3389/fnut.2021.712600 (PMC8631825; doi:10.3389/fnut.2021.712600)

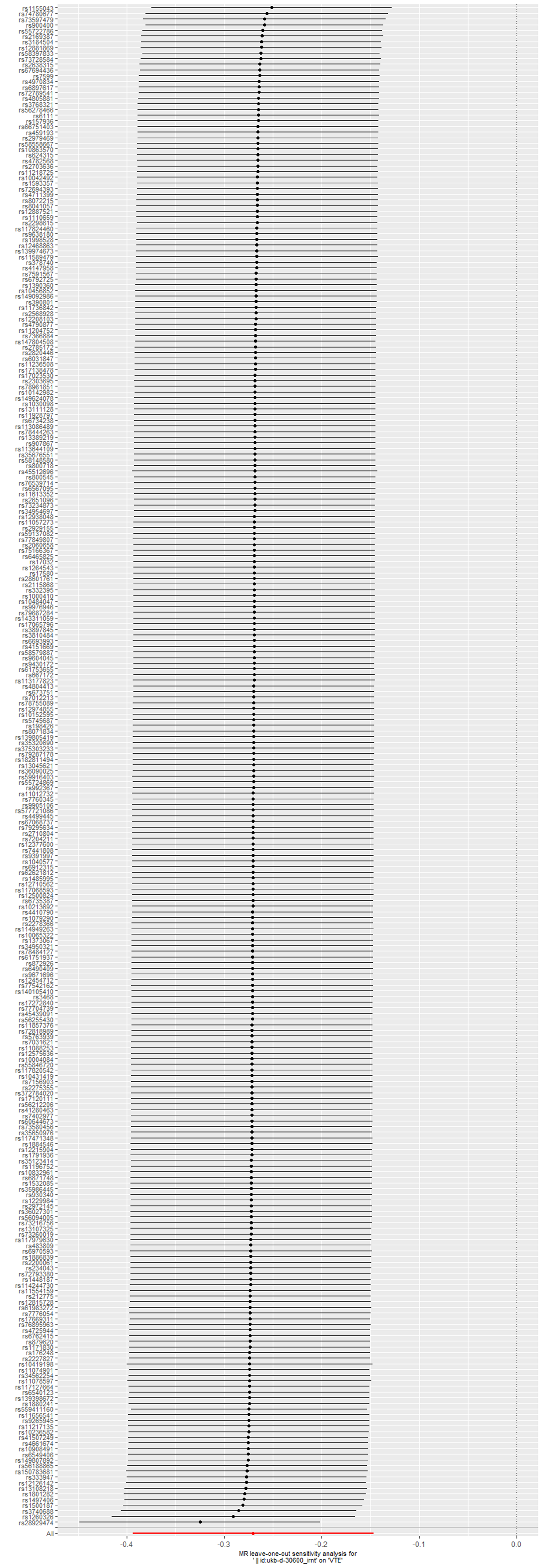

Supplement: Supplementary Figures 23–26 — Leave-one-out analysis of serum albumin, total protein, MUFAs, and ratios of MUFAs to the total fatty acid. [file Image_1.TIFF]

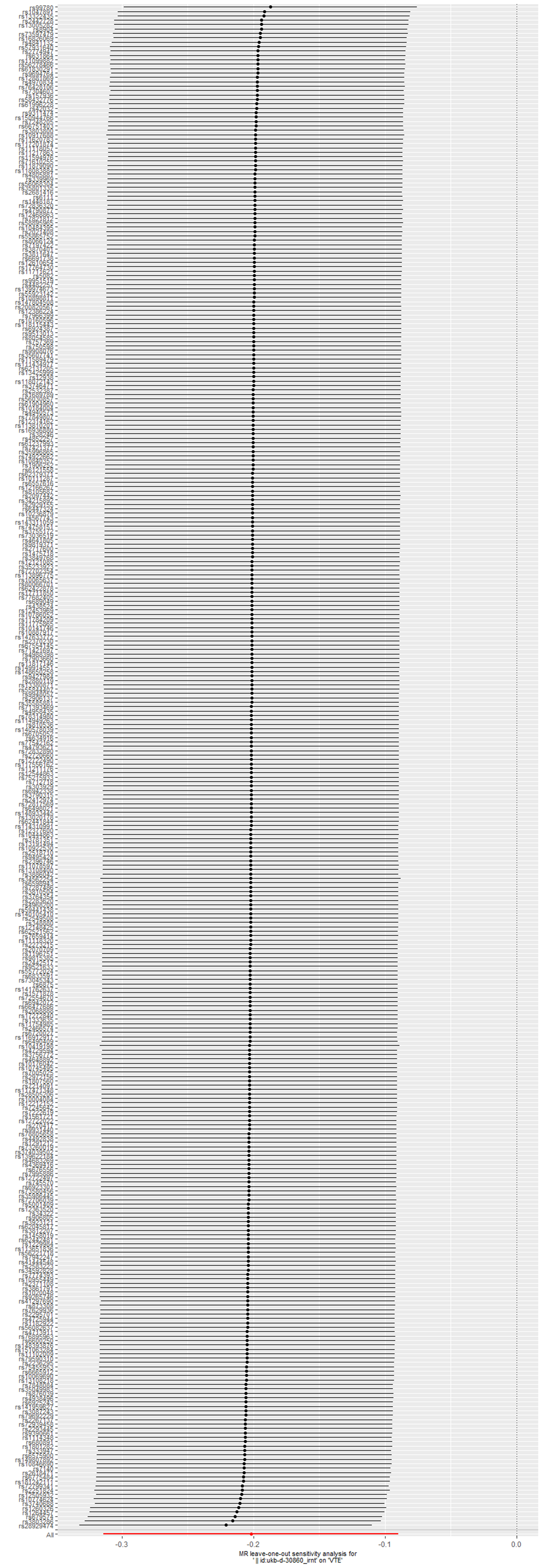

Supplement: Supplementary file 5 [file Image_2.TIFF]

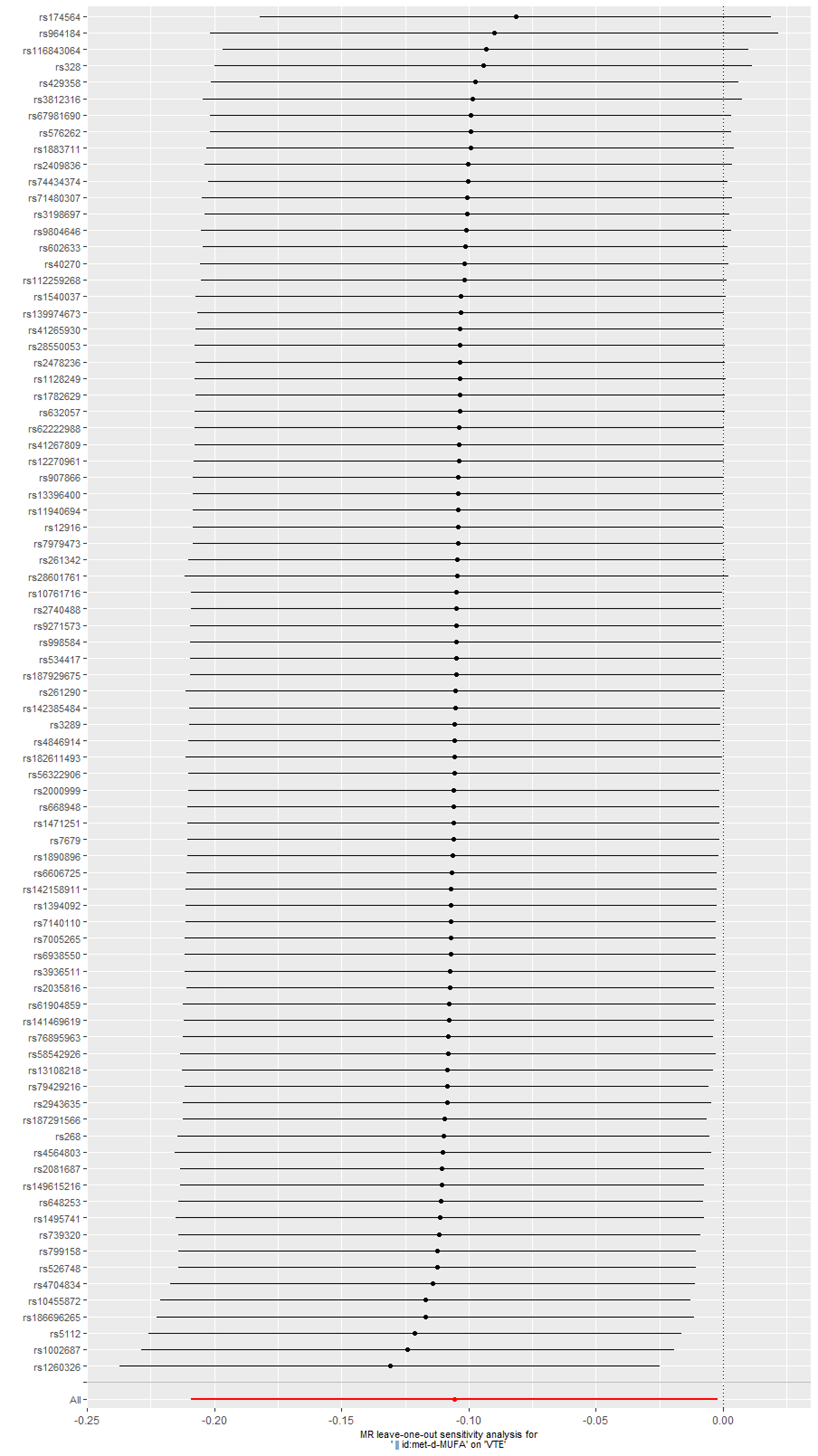

Supplement: Supplementary file 6 [file Image_3.TIFF]

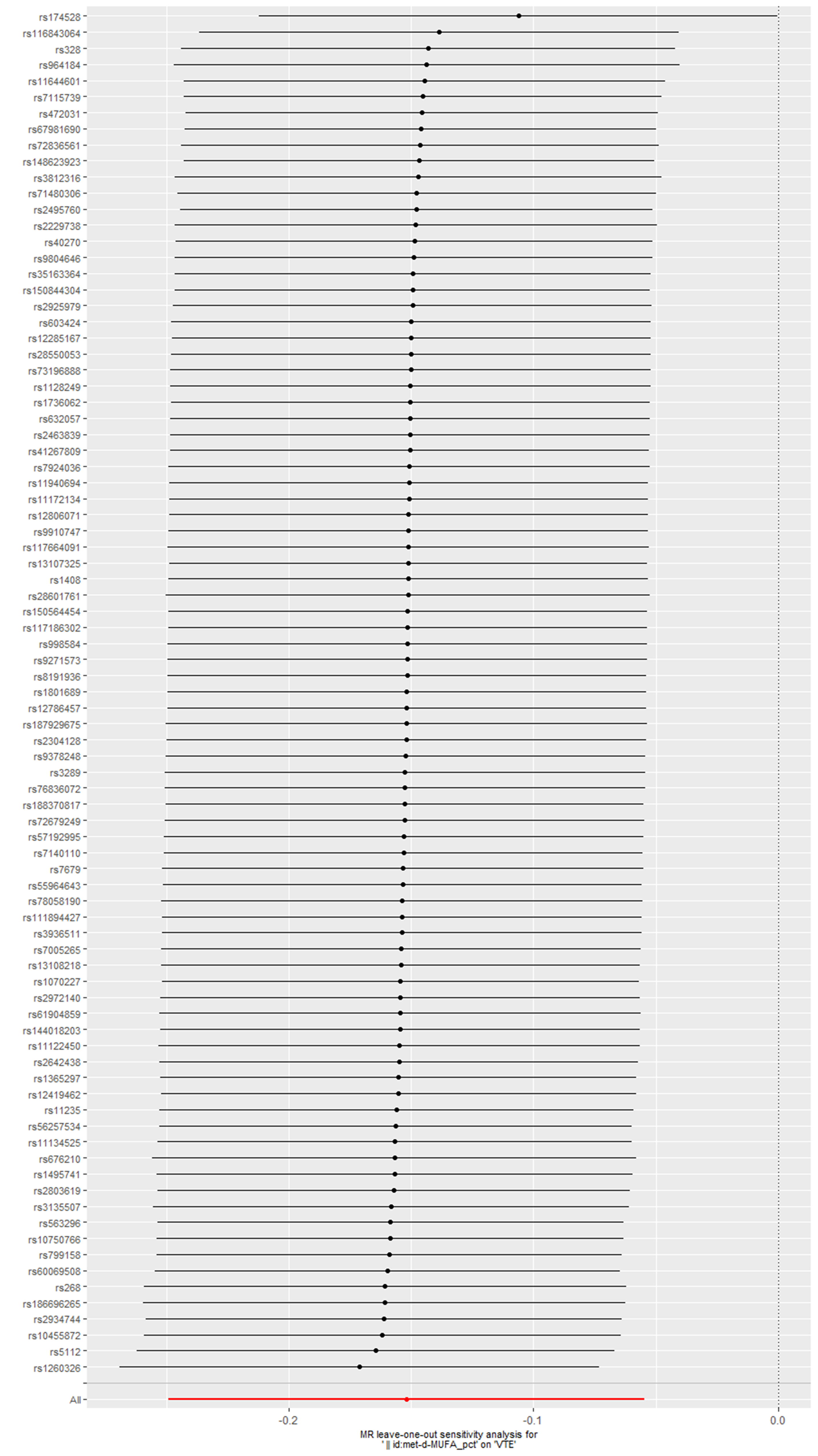

Supplement: Supplementary file 7 [file Image_4.TIFF]
